# Solitary Median Maxillary Central Incisor Syndrome: A Case Report of a Unique Dental Anomaly

**DOI:** 10.7759/cureus.58101

**Published:** 2024-04-12

**Authors:** Harikishan Kanani, Rutuja Patil, Monika Khubchandani, Ramakrishna Yeluri, Ruchika Pandey

**Affiliations:** 1 Pediatric Dentistry, Sharad Pawar Dental College and Hospital, Datta Meghe Institute of Higher Education & Research, Wardha, IND; 2 Orthodontics and Dentofacial Orthopedics, Sharad Pawar Dental College and Hospital, Datta Meghe Institute of Higher Education & Research, Wardha, IND

**Keywords:** paediatric dentistry, syndrome, central incisor, dental anomaly, solitary median maxillary central incisor syndrome

## Abstract

Solitary median maxillary central incisor (SMMCI) syndrome is complex and usually develops 35-38 days postconception during the intrauterine period. A noteworthy discovery is that just one central incisor in the maxillary alveolus, found exactly on the centerline, is present in both deciduous and permanent dentitions with other congenital anomalies. Around one in every 50,000 live babies exhibits this abnormality. This report describes the case of a 13-year-old female patient with SMMCI syndrome with a complaint about an unsightly appearance due to a single large upper front tooth. We underline the importance of increasing clinician awareness of SMMCI syndrome and the need for a multidisciplinary approach to its care.

## Introduction

Solitary median maxillary central incisor (SMMCI) syndrome is complex and usually develops 35-38 days postconception during the intrauterine period. It is characterized by a constellation of developmental abnormalities that mainly impact the maxilla’s midline components and are frequently caused by unknown etiological factors [1]. SMMCI syndrome was initially identified by Hall et al. in 1997. Their findings found that around one in every 50,000 live babies exhibited this abnormality, showing an affinity for the female gender [2,3].

The SHH gene’s missense mutation (I111F) at 7q36 may be linked to SMMCI, while the exact cause is unknown. This distinct developmental defect involves the head and body’s median line structures [4]. Difficulties in developing the upper jaw and its entire dental apparatus, particularly affecting the central incisor tooth germ, are characteristic features of congenital pyriform aperture stenosis (PAS), median line stenosis, and choanal atresia. Furthermore, certain cases might also show coexisting brain abnormalities like holoprosencephaly (HPE). Significantly, SMMCI syndrome is associated with all instances of HPE; however, the relationship is not reciprocal [5].

A noteworthy discovery is that just one central incisor in the maxillary alveolus, found exactly at the centerline, is present in deciduous and permanent dentitions [1]. A high labial position, abnormalities in the palatal suture, an abrupt palatal arch with a prominent mid-palatal ridge, an absence of the labial frenulum and incisive papillae, and an arch-shaped upper lip with an indistinct philtrum are other features that characterize this disorder. Collectively, these unique features add to the disease’s clinical profile and help clinicians diagnose and treat it [6].

Several syndromes and disorders, such as CHARGE syndrome, VACTERL association, velocardiofacial syndrome, autosomal dominant HPE, ectodermal dysplasia, Duane retraction syndrome, Goldenhar syndrome, and oromandibular limb hypogenesis syndrome type 1, can also be interconnected with patients with a single maxillary central incisor. These interactions underscore the intricate nature of SMMCI and highlight the critical need for comprehensive clinical evaluation and multidisciplinary treatment for individuals impacted by it [7].

To properly characterize the unique tooth present, several key details must be noted. First, it is solitary, indicating the presence of a single central maxillary incisor [5]. Second, it is positioned medially, precisely at the midpoint of the maxillary alveolar process [5]. Suppose one central incisor tooth is on one side of the midline. In that case, it points to the fact that the corresponding tooth on the opposite side may have been lost due to disease or injury, or it may have been unable to progress further than the cellular stage, resulting in the resorption of the tooth germ [5]. Third, this unique tooth is located in the maxilla, the upper jaw, not the lower one [5]. Last, despite its unique crown structure, it remains classified as a central incisor, distinct from mesiodens or supernumerary teeth [1,5].

An emergency surgical intervention is necessary for choanal stenosis. A short height could necessitate growth hormone treatment. The SMMCI tooth is mostly a cosmetic issue that may be corrected or left untreated. The best course of action is to combine orthodontic, prosthodontic, and oral surgical therapy. Since the patient needs ongoing, comprehensive care, it is preferable to treat them with a multidisciplinary healthcare team to promote their well-being [5].

## Case presentation

**Figure 1:**
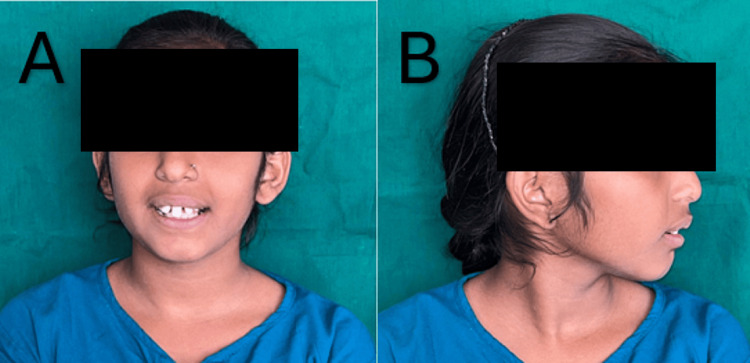
(A) Extraoral frontal view shows a symmetrical face with an unclear philtrum. (B) Extraoral lateral view shows a retrognathic chin and a convex profile.

A 13-year-old female patient presented to the Department of Pediatric and Preventive Dentistry at Sharad Pawar Dental College and Hospital in Wardha, India, with a complaint of an unsightly appearance caused by a single large upper front tooth. She was the only child in her household, and her mother reported no family history of genetic disorders. The patient’s birth history indicated an uneventful full-term delivery to healthy, unrelated parents (Figure 1).

The patient’s medical history indicated a healthy birth with no previous trauma or dental caries-related tooth loss. Facial examination revealed a symmetrical face, dolichocephalic head shape, and harmonious facial thirds. Characteristics of SMMCI syndrome, such as a high center section of the top lip and an unclear philtrum, were observed. Profile evaluation revealed a retrognathic chin, reduced nasolabial angle, and convex profile.

**Figure 2:**
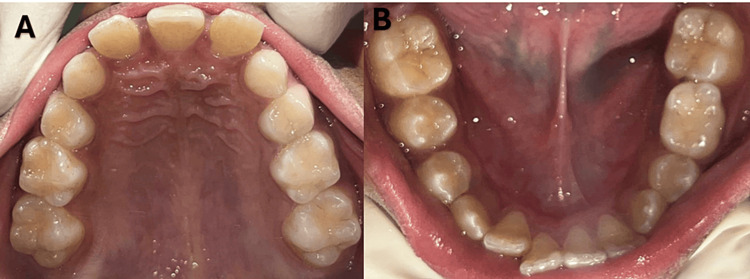
(A, B) Intraoral occlusal view of the maxillary arch and mandibular arch shows a mixed dentition stage. In the maxillary arch, a noticeable mid-palatal ridge is seen.

**Figure 3:**
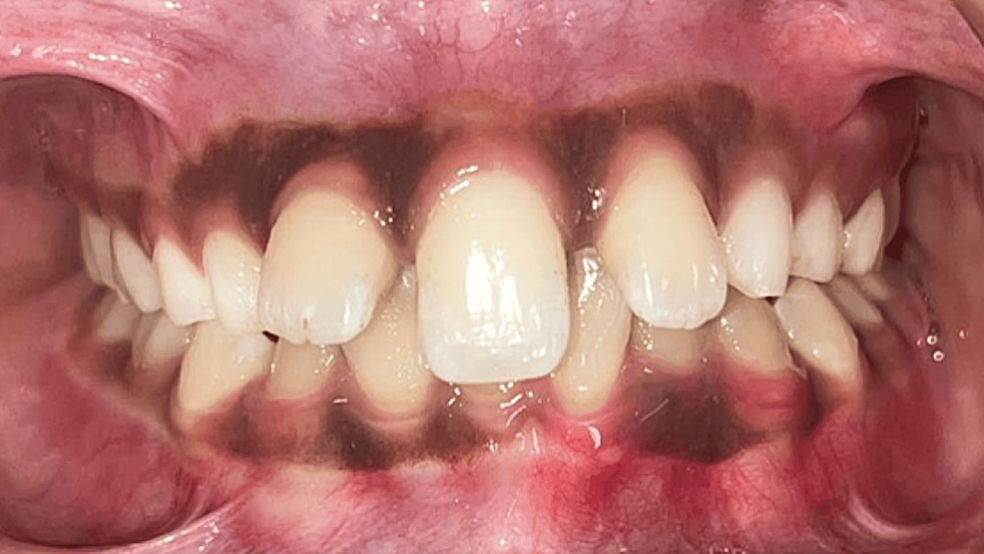
Intraoral frontal view in occlusion shows a single central incisor in the midline and absent the labial frenulum of the upper lip.

Intraoral examination showed a single massive maxillary central incisor precisely at the centerline, indicating a mixed dentition stage (Figure 2). Class I molar relationship angles were observed, with symmetrical maxillary and mandibular arches. A midpalatal vomerine ridge was noticeable, and the palate was V-shaped. The incisive papilla and labial frenulum of the upper lip were absent (Figures 2, 3).

**Figure 4:**
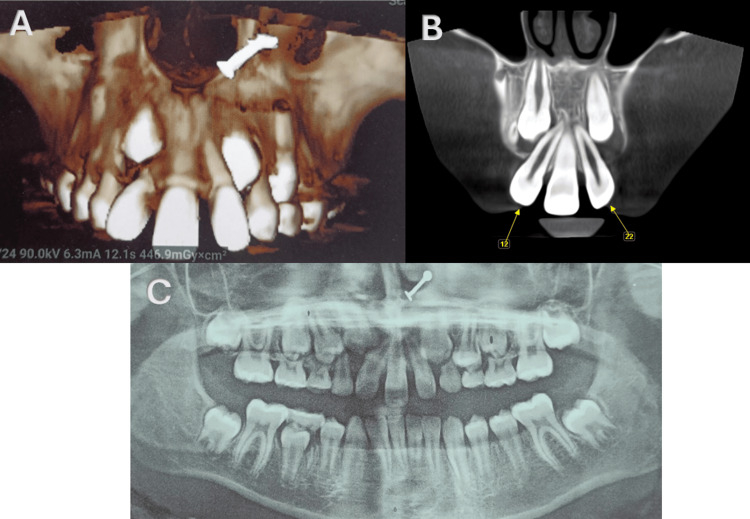
Cone beam computed tomography and orthopantomogram show the presence of a single permanent maxillary central incisor exactly in the midline.

The patient was found to have a symmetrical permanent single maxillary central incisor, as confirmed by both radiographic and clinical testing. No caries lesions or restorations were present. Additionally, the patient did not exhibit any other syndrome-related abnormalities, including intellectual incapacity (Figure 4).

Treatment alternatives

Since the patient’s primary concern was appearance, the only way to address this in SMMCI patients is to make room for the adjacent central incisor. When a patient reaches the right age, an orthodontist can utilize an expansion appliance to broaden the palate. This will allow for the SMMCI tooth to be shifted to any single side of the midline, which will free up room for the prosthesis to replace the contralateral missing central incisor. Following the establishment of space, fixed prosthodontic rehabilitation may be used to replace the incisor. The fixed prosthodontic treatment options include bridges, Maryland retainers, and single-tooth implants. For teenagers, fixed partial dentures or bridges are not recommended. Additionally, until alveolar growth is complete, a single-tooth implant is not advised since this might lead to an implant location that differs from neighboring natural teeth, which continue to grow during adolescence. In certain situations, Maryland retainers that need less dental preparation may be utilized; however, they frequently break and fail. The simplest course of treatment is to utilize a modified removable orthodontic retainer with an artificial central incisor until the growth is finished and an implant or bridge can be employed. In addition to prosthodontic rehabilitation of lost teeth, the SMMCI tooth can be reshaped using a labial veneer once growth has finished (17-18 years of age) to establish the anatomical form of the proper size and shape.

## Discussion

The maxillary central incisor’s distinct shape and location indicate the unusual developmental abnormality known as the SMMCI tooth. According to Hall, if there is solely one central incisor tooth on one side of the midline, it points to the fact that the corresponding tooth on the opposite side may have been lost due to disease or injury, or it may have been unable to progress further than the cellular stage, resulting in the resorption of the tooth germ. This syndrome results from an interruption in normal cell division at an essential phase for structures at the midline, usually about gestation days 37 or 38. As a result, rather than developing two distinct central incisors, the distal half of these teeth fuse to form a single tooth. This fusion process involves mesenchymal condensations and inductive epithelium, ultimately establishing a single, centrally positioned tooth in the maxilla [5].

The precise pathogenic or genetic abnormality causing SMMCI remains unknown. On the other hand, some data suggests that a mutation in the sonic hedgehog gene on the chromosome 7q long arm may cause isolated SMMCI [8]. In individuals with SMMCI, Dolan et al. and Aughton et al. reported deletions in portions of chromosome 18 (18p), while Masuno et al. found deletions in the terminal region of chromosome 7q [9-11].

SMMCI may manifest alone or in conjunction with other morphologic abnormalities, including hypotelorism, a blurred philtrum, the loss of the upper lip’s frenulum, a vomerine ridge, nasal obstruction, or septal deviation [12]. Scott was the first to disclose the involvement of SMMCI, detailing an isolated discovery of a female who had a single median maxillary central incisor [13]. Fulstow outlined a scenario of SMMCI where the person exhibited microcephaly, scoliosis, low height, and congenital heart disease in addition to having a single central incisor [14].

Studies that have been published in the literature have not discovered any link between SMMCI and systemic alterations. Two examples of SMMCI in individuals with average height were described by Wesley et al. [15]. At the same time, three cases of SMMCI without systemic involvement and development deficits were reported by Cho and Drummond [16]. SMMCI’s clinical relevance stems from its correlation with HPE, a fatal condition when fully manifested. The offspring of individuals with isolated SMMCI are susceptible to HPE because SMMCI is believed to be the mildest type of HPE [17].

Numerous congenital nasal cavity abnormalities, including nasal PAS, mid-nasal stenosis, and choanal atresia, may be linked to SMMCI [2,5]. Four of the six individuals Arlis and Ward assessed had SMMCI [17]. Every patient in the research had nasal pyriform aperture congenital stenosis. All 21 individuals with single maxillary central incisor syndrome had a history of congenital nasal obstruction, according to research by Hall. Of these, eight had intranasal stenosis, and seven had choanal atresia. Similarly, Lo et al. found that 63% of those with nasal pyriform aperture congenital stenosis also had SMMCI. These findings highlight a potential connection between congenital nasal obstruction and SMMCI, indicating a possible similar pathophysiology or genetic foundation that necessitates additional research [5,18].

Deviations in craniofacial morphology and neurocranial size and form have been linked to SMMCI. Tabatabaie et al. assessed the neurocranial and craniofacial morphology of thirteen children with SMMCI using cephalometric analysis and profile radiographs. They found a considerable reduction in the size of the neurocranium, maxillary prognathic, maxillary inclination, mandibular prognathic, and mandibular incisor inclination in SMMCI [19].

While dentists primarily report isolated cases of SMMCI, instances of SMMCI accompanied by systemic abnormalities have been documented by both medical and dental professionals. As a result, very few examples of SMMCI as an isolated abnormality have been published in the literature. SMMCI should be diagnosed as soon as possible since it might indicate other, more severe congenital problems. A comprehensive approach to treatment and a multidisciplinary team consisting of pediatric dentists, prosthodontists, and orthodontists are imperative for the effective dental management of individuals with SMMCI [7].

## Conclusions

SMMCI syndrome is a rare developmental condition characterized by a single central incisor in the maxilla as well as other dental, craniofacial, and systemic defects. Early identification and extensive evaluation are critical for effective management and optimal results. Through this case report, we underline the importance of increasing clinician awareness of SMMCI syndrome and the need for a multidisciplinary approach to its care. More study is needed to understand the underlying cause and enhance treatment techniques for this rare disease.
